# FFF‐VMAT for SBRT of lung lesions: Improves dose coverage at tumor‐lung interface compared to flattened beams

**DOI:** 10.1002/acm2.12764

**Published:** 2019-12-20

**Authors:** Damodar Pokhrel, Matthew Halfman, Lana Sanford

**Affiliations:** ^1^ Department of Radiation Medicine Medical Physics Graduate Program University of Kentucky Lexington KY USA

**Keywords:** FFF‐beam, lung SBRT, noncoplanar VMAT, RTOG‐0915, single‐dose

## Abstract

**Purpose:**

To quantify the differences in dosimetry as a function of ipsilateral lung density and treatment delivery parameters for stereotactic, single dose of volumetric modulated arc therapy (VMAT) lung stereotactic body radiation therapy (SBRT) delivered with 6X flattening filter free (6X‐FFF) beams compared to traditional flattened 6X (6X‐FF) beams.

**Materials/methods:**

Thirteen consecutive early stage I–II non‐small‐cell‐lung cancer (NSCLC) patients were treated with highly conformal noncoplanar VMAT SBRT plans (3–6 partial arcs) using 6X‐FFF beam and advanced Acuros‐based dose calculations to a prescription dose of 30 Gy in one fraction to the tumor margin. These clinical cases included relatively smaller tumor (island tumors) sizes (2.0–4.2 cm diameters) and varying average ipsilateral lung densities between 0.14 g/cc and 0.34 g/cc. Treatment plans were reoptimized with 6X‐FF beams for identical beam/arc geometries and planning objectives. For same target coverage, the organs‐at‐risk (OAR) dose metrics as a function of ipsilateral lung density were compared between 6X‐FFF and 6X‐FF plans. Moreover, monitor units (MU), beam modulation factor (MF) and beam‐on time (BOT) were evaluated.

**Results:**

Both plans met the RTOG‐0915 protocol compliance. The ipsilateral lung density and the tumor location heavily influenced the treatment plans with 6X‐FFF and 6X‐FF beams, showing differences up to 12% for the gradient indices. For similar target coverage, 6X‐FFF beams showed better target conformity, lower intermediate dose‐spillage, and lower dose to the OAR. Additionally, BOT was reduced by a factor of 2.3 with 6X‐FFF beams compared to 6X‐FF beams.

**Conclusion:**

While prescribing dose to the tumor periphery, 6X‐FFF VMAT plans for stereotactic single‐dose lung SBRT provided similar target coverage with better dose conformity, superior intermediate dose‐spillage (improved dose coverage at tumor interface), and improved OAR sparing compared to traditional 6X‐FF beams and significantly reduced treatment time. The ipsilateral lung density and tumor location considerably affected dose distributions requiring special attention for clinical SBRT plan optimization on a per‐patient basis. Clinical follow up of these patients for tumor local‐control rate and treatment‐related toxicities is in progress.

## 1 INTRODUCTION

Due to the recent technological advances in lung stereotactic body radiation therapy (SBRT) treatments and reported comparable tumor local‐control rates,^1^, ^2^, ^3^, ^4^, ^5^, ^6^, ^7^single‐dose lung SBRT has become a viable treatment option for peripherally located lung lesions for medically inoperable early‐stage nonsmall‐cell lung cancer (NSCLC) patients.^8^, ^9^, ^10^ Additionally, there has been growing interest in the clinical use of flattening filter‐free (FFF) beams to deliver lung SBRT treatment.^11^, ^12^, ^13^, ^14^, ^15^ FFF‐beams have much higher dose rates compared to flattened beams and consequently reduce beam on time significantly. This results in better patient comfort (less time on the table), reduced dose delivery uncertainty due to intrafraction motion, and reduced out‐of‐field dose due to head scatter and electron contamination.^15^

Combining FFF‐beams with volumetric modulated arc therapy (VMAT) 16, 17 results in even greater treatment efficiency for complex lung SBRT plans compared to historically used 8–15 noncoplanar fixed fields or several noncoplanar dynamic conformal arc (DCA) plans. Linac‐based intensity modulated radiation therapy (IMRT), helical TomoTherapy or optimized robotic CyberKnife treatments significantly prolong SBRT treatment time, comparatively.^18^, ^19^, ^20^, ^21^

However, FFF‐beams have different beam characteristics compared to flattened beams as mentioned earlier.^15^ These include a nonuniform beam profile, reduced mean energy, and differing penumbra at depth. Given these distinct physical characteristics of FFF‐beams, a few previous researchers have studied the dosimetric advantages of FFF‐ vs FF‐beams in SBRT lung treatments.^11^, ^12^, ^13^, ^14^ The majority of the previous studies showed similar target coverage and clinically insignificant dose differences to the organs‐at‐risk (OAR) for both beam types, however, FFF‐beams resulted in much faster treatment times.

While using flattened beams, rings of underdosing around the tumor and at lung tissue interfaces have been previously reported by Chetty et al. 22, 23In the most recent study by Vassiliev et al., 24 it was demonstrated that 6X‐FFF beams can mitigate dose loss at the tumor‐lung periphery due to low energy secondary electron dose buildup at the interface. Their experiment was conducted in a phantom comprised of a chest wall, lung tissue, and spherical tumors of 1, 3, and 5 cm diameters. Three lung densities of 0.1, 0.2, and 0.26 g/cc were considered. Treatment plans were generated using 7‐coplanar static‐fields with 6X‐FFF and 6X‐FF beams for a total dose of 50 Gy in five fractions prescribed to the tumor center. EGSnrc Monte Carlo, anisotropic analytical algorithm (AAA), and Acuros‐based dose calculations were performed on a Truebeam linear accelerator. Monte Carlo dose distributions revealed that 6X‐FF beams underdosed the periphery of the tumor by about 2 Gy, most noticeably for smaller tumors and lower lung densities, while prescribing a point dose at the tumor center. Their study exposed the true difference between these delivery approaches; however, current clinical practice for lung SBRT treatment is to prescribe dose to the tumor periphery. Therefore, at least 95% of the PTV should receive the prescribed dose with a 120–130% hot spot at the tumor center. Furthermore, daily clinical practice heavily relies on complex noncoplanar beam geometries including VMAT planning for lung SBRT treatments that could potentially smear the dose buildup at the periphery of the tumor.

For our single‐dose (30 Gy prescription to the tumor margin) lung SBRT treatments we use highly conformal noncoplanar VMAT (most commonly with ± 5°–10° table kicks) plans that delivers 3–6 partial arcs utilizing a 6X‐FFF (1400 MU/min) beam on a Truebeam linac. Single‐dose lung SBRT is an extreme form of hypofractionation used in our clinic for extracranial lesions where the dose calculation could potentially suffer by tumor size, tumor location, and the presence of inhomogeneities in the lung. The aforementioned study by Vassiliev et al.^24^ prompted us to examine the dosimetric behaviors of 6X‐FFF verses 6X‐FF VMAT plans while prescribing clinically realistic doses to the tumor periphery and using complex treatment beam/arc geometries in the clinical cases. Our real clinical cases include relatively smaller tumor sizes of 2.0–4.2 cm diameter (mostly island tumors) and varying ipsilateral lung densities between 0.14 g/cc and 0.34 g/cc, on average.

Therefore, herein we have retrospectively evaluated the dosimetry of 13 consecutive lung SBRT patients who underwent a stereotactic, single‐dose treatment of 30 Gy using noncoplanar VMAT plans with 6X‐FFF beams, and reoptimized those plans with 6X‐FF beams. The original clinical 6X‐FFF and 6X‐FF plans were compared via lung SBRT protocol^8^ compliance criteria for target conformity, gradient indices, and dose to the OAR. Additionally, dosimetric behaviors as a function of average ipsilateral lung density and improvement of treatment efficiency of 6X‐FFF plans were reported.

## 2 MATERIALS AND METHODS

### 2.A Patient characteristics

After obtaining an institutional review board (IRB) approval from our institution, 13 peripherally located tumors in early stage I‐II NSCLC patients who received a single dose of 30 Gy via lung SBRT were evaluated. Tumor characteristics are summarized in Table 1 which include tumor location, mean values of ipsilateral lung Hounsfield units (HU), and the corresponding average lung density. In this cohort of patients, the mean planning target volume (PTV) is 13.0 ± 12.2 cc (range 4.3–41.1 cc), and the corresponding average tumor diameter is 2.7 ± 0.7 cm (range 2.0–4.2 cm). The ipsilateral lung excludes the ITV and the mean density value ranges from 0.14 to 0.34 gm/cc.

**Table 1:** Characteristics of lung SBRT patients included in this study. Prescription dose was 30 Gy in one fraction.

Patient no.	Tumor location	ITV (cc)	PTV (cc)	PTV diameter, *d* (cm)	Ipsilateral‐lung minus ITV	
					Mean HU	Mean density (g/cc)
1	Midcentral, left lung	0.2	5.0	2.11	−761	0.24
2	Midcentral, left lung	0.3	5.1	2.12	−751	0.25
3	Upper lobe, left lung	0.7	6.4	2.28	−666	0.33
4	Lower lobe, right lung	0.7	8.2	2.48	−713	0.29
5	Upper lobe, left lung	10.1	41.1	4.22	−668	0.33
6	Midcentral, right lung	1.1	10.7	2.71	−858	0.14
7	Upper lobe, left lung	0.3	4.3	2.00	−716	0.28
8	Midcentral, right lung	13.6	37.5	4.09	−781	0.22
9	Upper lobe, left lung	0.8	5.2	2.13	−791	0.21
10	Upper lobe, left lung	1.4	11.0	2.73	−662	0.34
11	Upper lobe, left lung	2.1	14.8	3.00	−789	0.21
12	Midcentral, right lung	2.5	14.4	3.00	−823	0.18
13	Upper lobe, left lung	0.5	5.8	2.20	−661	0.34

ITV, Internal target volume; PTV, planning target volume; SBRT, stereotactic body radiation therapy.

### 2.B Imaging and target delineation

The patients were immobilized using Body Pro‐Lok^TM^ platform (CIVCO system, Orange City, IA) in the supine position with their arms above their head using an armrest and abdominal compression. The free‐breathing planning 3D‐CT simulation was performed on a GE Lightspeed 16 slice CT scanner (General Electric Medical Systems, Waukesha, WI) with 512 × 512 pixels at 2.5 mm slice thickness in the axial helical mode. Following the 3D‐CT scan, all patients underwent a respiration‐correlated 4D‐CT scan using the Varian RPM System (version 1.7) in the same position. The 4D‐CT images were reconstructed in ten equally spaced phase bins using an Advantage 4D Workstation (General Electric Medical Systems, San Francisco, CA), where the maximum intensity projection (MIP) images were generated. The regular 3D‐CT and the MIP images were imported into Eclipse TPS (version 13.6, Varian Medical Systems, Palo Alto, CA) and coregistered for target delineation. Internal target volume (ITV) was delineated on the MIP images of the 4D‐CT and mapped to 3D‐CT images for dose calculation. The PTV was generated by adding a uniform 5 mm margin to the ITV per RTOG 0915 guidelines.^8^ The relevant critical structures such as bilateral lungs excluding the ITV (normal lung), spinal cord, ribs, heart, big vessels, esophagus, and skin were delineated on the 3D‐CT images for dose reporting.

The average ipsilateral lung density was calculated using the following equation: ρ_lung_ = ρ_water_ (1.0 + avg. HU/1000). Average HU of the ipsilateral lung (excluding ITV) was obtained from the Eclipse TPS using ipsilateral lung contour for each patient.

### 2.C Clinical 6X‐FFF plans and treatment delivery

For each patient, highly conformal, clinically optimal VMAT SBRT plans were generated in Eclipse TPS using 3–6 (median, 4) partial noncoplanar arcs (±5°–10°, couch kicks for arcs) on a Truebeam linear accelerator (Varian Palo Alto, CA) in Eclipse TPS consisting of standard millennium multileaf collimators (MLC), and 6 MV‐FFF (1400 MU/min) beams. The isocenter was placed at the geometric center of the PTV. These partial noncoplanar arcs had an arc‐length of approximately 200°–220° gantry rotation, and the collimator angles (between 30° and 135°) were manually optimized to reduce the MLC tongue‐and‐groove dose leakage throughout the arc rotation on a per‐patient basis. Jaw tracking was utilized during plan optimization to further minimize out‐of‐field leakage. The prescription dose was 30 Gy in one fraction so that at least 95% of the PTV received 100% of the prescribed dose and all hot spots (120–130%) fell within the ITV. All clinical treatment plans were calculated using the TPS with the advanced Acuros‐XB (version 13.6.0) algorithm^25^, ^26^, ^27^, ^28^ on the 3D‐CT images with heterogeneity corrections, 1.25 mm calculation dose grid size, and using the photon optimizer (PO) MLC algorithm. The dose to medium reporting mode was used. All clinical plans were inversely optimized using varying gantry rotation speed, dose rate, and MLC positions. Planning objectives followed RTOG‐0915 requirement (Arm 1).

The patient‐specific quality assurance (QA) of these plans was performed by delivering VMAT SBRT plans on an Octavius phantom (PTW, Freiburg, Germany). All VMAT QA plans were delivered on Truebeam before the patient start date. The measured cumulative 2D dose plan was compared with the computed dose distributions calculated on the Octavius QA phantom plan in Eclipse TPS. Upon completion of dose delivery, data were analyzed with Octavius MEPHYSTO Navigator (VeriSoft Patient Plan Verification, Version 6.3, PTW) using the clinical gamma passing rate criteria of 3%/3mm maximum dose difference and distance‐to‐agreement (DTA) with a 10% threshold. The average VMAT QA pass rate was 97.6 ± 2.7%. All patients were treated with CBCT‐guided imaging. Patient setup prior to single‐dose lung SBRT was performed using an in‐house SBRT/IGRT protocol by coregistering pretreatment cone‐beam CT with the planning CT scans. The patient setup, tumor matching online cone‐beam CT scan, and treatment delivery were monitored and verified by the treating physician and physicist.

### 2.D Reoptimized 6X‐FF plans

For comparison, the standard clinical 6X‐FFF plans were reoptimized in Eclipse TPS using identical arc geometries while utilizing 6X‐FF beams. The same collimator rotation and jaw tracking approaches were applied. For these VMAT plans, optimization objectives matched the 6X‐FFF plans, and all 6X‐FF plans were normalized identically to the clinical 6X‐FFF plans as described above.

### 2.E Plan quality comparison

The original clinical 6X‐FFF and reoptimized 6X‐FF plans were compared in terms of their protocol compliance criteria, target conformity, gradient indices, and dose to OAR. Delivery efficiency was also evaluated. The DVHs of all treatment plans were evaluated per RTOG‐0915 high and intermediate dose spillage dosimetry parameters: 8

- Conformity index, CI: ratio of prescription isodose volume to the PTV. CI less than 1.2 is highly desirable; CI = 1.2–1.5, acceptable with minor deviations.
- Gradient index, GI: ratio of 50% prescription isodose volume to the PTV. GI has to be smaller than 3–6, depending on the PTV.
- Maximum dose at any point 2 cm away from the PTV margin in any direction, D_2cm_: D_2cm_ has to be smaller than 50–70%, depending on the PTV size.
- Percentage of normal lung receiving dose equal to 20 Gy or more, V_20Gy_: V_20Gy_ should be less than 10% per protocol, V_20Gy_ <15% is acceptable with minor deviations. V_20Gy_ is for total lungs minus the ITV.
- Heterogeneity index, HI: Dmax/prescribed dose was used to evaluate the dose heterogeneity within the PTV.
- Gradient distance, GD: GD is the average distance from 100% prescribed dose to 50% prescribed dose which indicates how sharp the dose falls off. The gradient distance (GD) is used to evaluate dose sparing to normal lung volume.
- Total number of monitor units, MU.
- Modulation factor, MF: ratio of total number of MU to the prescription dose in cGy.
- Beam‐on time, BOT.

Furthermore, all clinical 6X‐FFF and reoptimized 6X‐FF plans were evaluated for the relative volume of normal lung receiving 5 Gy and 10 Gy doses, mean lung dose (MLD) and maximum dose received by 1000 cc of lungs. The dose to spinal cord (maximum and 0.35 cc), heart (maximum and 15 cc), and esophagus (maximum and 3 cc) were analyzed. Since all of these cases had peripheral lung lesions, the doses to ribs (maximum and 1 cc) and skin (maximum and 10 cc) were also evaluated. Moreover, intermediate dose spillage as a function of ipsilateral lung density was investigated.

A paired matched two tail Student’s t‐test was used to compare the difference with a cutoff *P* value < 0.05 indicating statistical significance. RTOG dose limits for maximum doses to spinal cord <14.0 Gy, heart <22.0 Gy, esophagus <15.4 Gy, maximum dose and dose to 1 cc of ribs, <30.0 Gy and < 22.0 Gy, maximum dose and 10 cc of skin <26.0 Gy and <23.0 Gy, respectively were used for plan evaluation per single‐fraction lung SBRT protocol (see Arm 1).

## 3 RESULTS

### 3.A Dosimetric parameters

Both plans achieved the RTOG‐0915 protocol compliance and were clinically acceptable for stereotactic treatment. The 6X‐FFF plans had the advantage of providing less intermediate dose‐spillage (see GI, D2cm, GD, significant *P*‐values in Table 2) compared to traditional 6X‐FF plans. Statistically significant *P*‐values are in bold (see Table 2). In general, mean dose to the ITV was similar between the plans, however, the maximum and minimum doses to the ITV were significantly higher (see *P* values in Table 2) for 6X‐FF plans compared to 6X‐FFF plans.

**Table 2:** Plan quality evaluation for clinical 6X‐FFF and 6X‐FF (replanned) plans for all 13 VMAT lung SBRT patients.

Target volume	Parameters	6X‐FF	6X‐FFF	*P*‐value
PTV	CI	1.09 ± 0.11 (0.98–1.39)	1.07 ± 0.08 (0.98–1.24)	n. s.
	GI	5.8 ± 1.3 (3.92–8.11)	5.5 ± 1.1 (3.81–7.23)	***P = 0.001***
	D2cm (%)	50.0 ± 5.5 (38.8–58.8)	48.0 ± 4.7 (37.8–55.1)	***P = 0.001***
	GD (cm)	1.1 ± 0.2 (0.79–1.52)	1.0 ± 0.2 (0.77–1.37)	***P = 0.001***
	HI	1.24 ± 0.1 (1.13–1.36)	1.20 ± 0.1 (1.10–1.25)	***P = 0.002***
ITV	D_min_ (Gy)	32.8 ± 1.6 (28.7–34.5)	32.3 ± 1.3 (28.9–33.8)	***P = 0.04***
	D_max_ (Gy)	37.0 ± 2.0 (33.9–40.9)	35.9 ± 1.5 (33.1–37.9)	***P = 0.002***
	D_mean_ (Gy)	35.2 ± 1.8 (32.9–38.8)	34.4 ± 1.2 (32.5–36.3)	n. s.

ITV, internal target volume; n. s., not significant; PTV, Planning target volume; SBRT, stereotactic body radiation therapy.

The statistical significance at *P* < 0.05 are bold.

**Figure 1:**
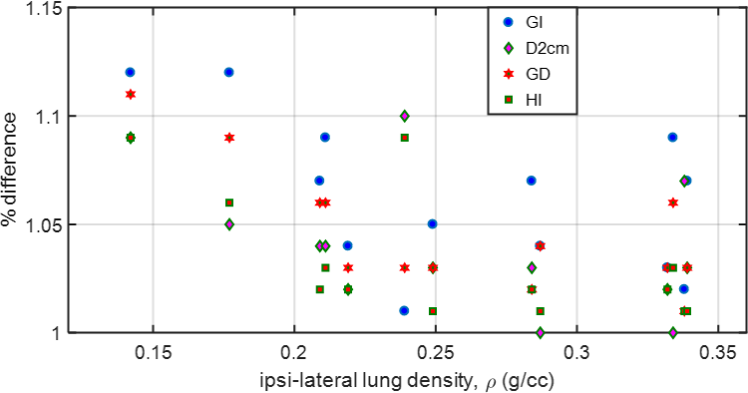
Scatter plot: Ratio between 6X‐FF and 6X‐FFF plans for GI, D2cm, GD and HI as a function of average ipsilateral lung density for all 13 patients. Up to 12% higher values were observed for GI and GD with lower ipsilateral lung density with 6X‐FF plans. In general, as the ipsilateral lung density decreases, the deviation in the gradient indices between the plans increases.

The variations of gradient and heterogeneity indices between 6X‐FFF and 6X‐FF plans as a function of the ipsilateral lung density are plotted in Fig. 1. Traditional 6X‐FF beams systematically overpredicted gradient indices (up to 12% higher) as a function of lower ipsilateral lung density suggesting that it could potentially deliver higher intermediate dose‐spillage to the nontarget tissues compared to 6X‐FFF beams.

**Figure 2:**
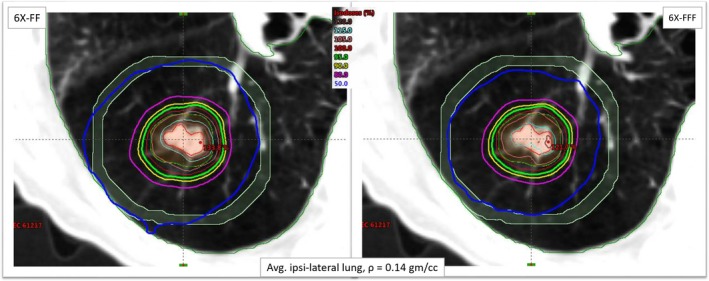
Comparison of dose distributions in the axial view for patient #6 (lowest average ipsilateral lung density of 0.14 gm/cc) with both 6X‐FFF (right panel) and 6X‐FF (left panel) beams. The red contour represents the ITV, orange represents the PTV (10.7 cc), green represents the 95% isodose line that encompasses the PTV and the blue line represents the 50% isodose‐spillage (much tighter with 6X‐FFF beam). The tumor was located in the middle of the right lung. The viewing plane intersection shows the isocenter location. The light blue ring was generated to calculate D_2cm_ around the target volume. ITV, Internal target volume; PTV, planning target volume.

Figure 2 shows an example case of radiosurgical dose distributions in the axial view through the isocenter plane for a lung SBRT patient (patient #6) with 6X‐FFF (right panel) and 6X‐FF (left panel). This patient presented with the lowest average ipsilateral lung density in the cohort. The PTV was 10.7 cc (2.71 cm diameter) and located in the midcentral right lung (island tumor). For example, in this case, the CI, HI, D_2cm_, GI, GD, and V20 were 1.24 vs 1.39, 1.25 vs 1.36, 53.8% vs 58.8%, 7.23 vs 8.11, 1.37 cm vs 1.52 cm and 0.6% vs 0.7%, 6X‐FFF vs 6X‐FF plan, respectively; all plan evaluation parameters were in favor of the 6X‐FFF beams. Major dosimetric differences were observed in the values of intermediate dose‐spillage and hot spots (tumor heterogeneity) with 6X‐FF plans compared to 6X‐FFF plans. In addition, the absolute dose differences to the OAR were up to 1.0 Gy with 6X‐FF plans, although, the OAR dose differences were shown to be clinically insignificant. However, the difference in the treatment delivery time was improved by a factor of 2.5 with 6X‐FF plans (17.65 min) vs 6X‐FFF (7.21 min) plans.

**Figure 3:**
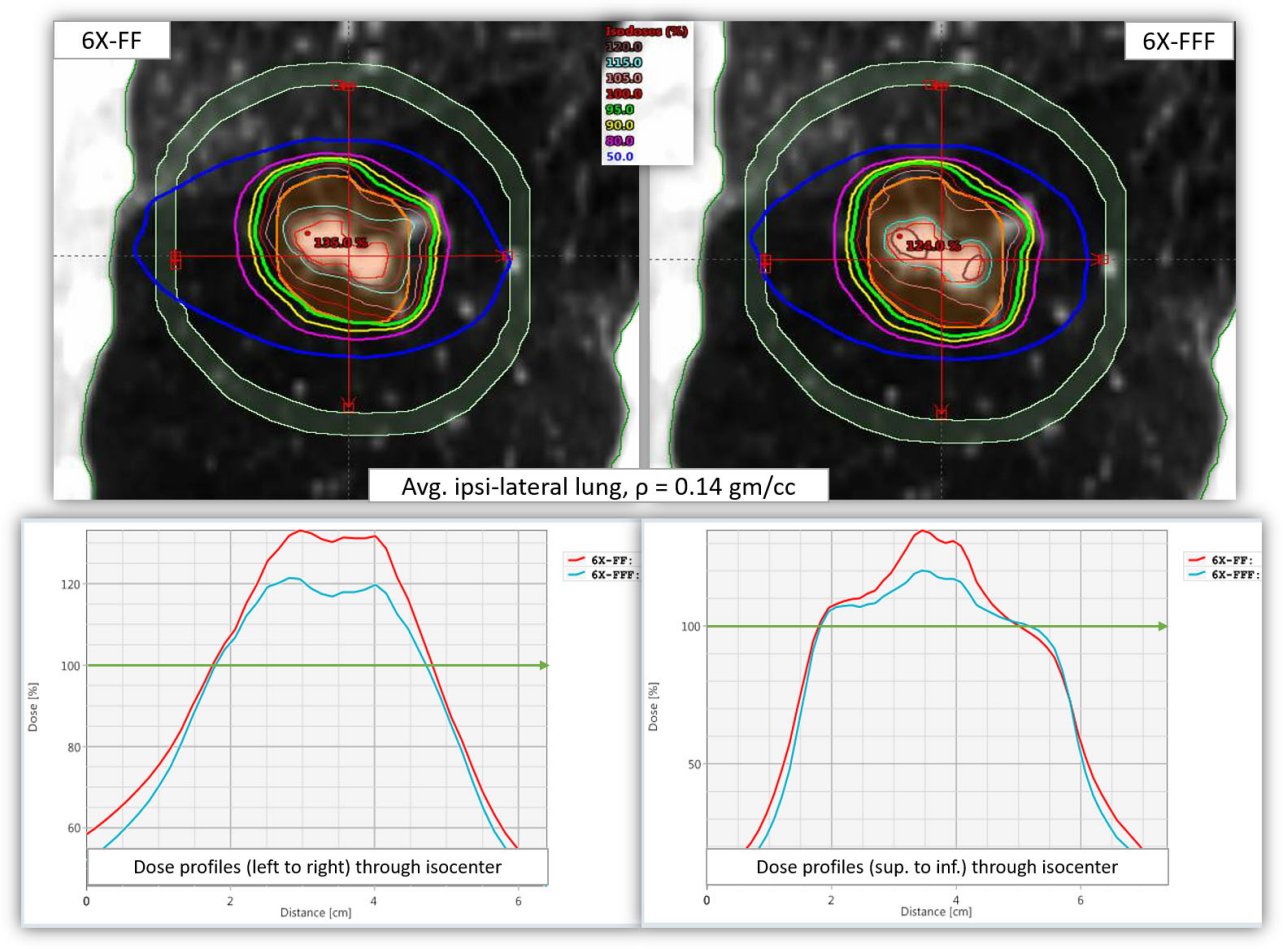
Upper panel: Isodose distributions from 6X‐FF (left) and 6X‐FFF (right) plans in coronal views for patient #6 as shown in Figure 1. Lower panel: Corresponding radial dose profiles drawn through the isocenter. Bottom left shows the dose profiles from left to right and the bottom right shows the dose profiles from superior to inferior directions (within D2 cm). The green lines on each graph show that both plans were normalized to get 95% of the target to receive 100% of the prescription dose. For identical target coverage, the ITV and the out‐of‐field doses (intermediate dose‐spillage and dose to the OAR) were higher with 6X‐FF beams. ITV, Internal target volume; OAR, organs‐at‐risk.

We calculated the dose profiles as a function of distance through the isocenter position (from right to left and superior to inferior within D2cm) for patients with the lowest average ipsilateral lung density in this cohort. Figure 3 clearly shows that the dose distributions heavily depend on the lung density between the two plans. For similar target coverage, the 6X‐FFF beams gave tighter dose distributions (less intermediate dose‐spillage and more conformal dose distributions), relatively small hot spots and fewer dose to the OAR compared to traditional 6X‐FF beams. This is due to the low lung density where the secondary electrons that have a relatively longer range in lung parenchyma with 6X‐FF (average photon energy 1.75 MeV) beam compared to clinical 6X‐FFF beam (average photon energy 1.28 MeV). 12 In addition, the imbalance of the rates of secondary electron production in lung parenchyma and the tumor itself caused dose buildup differences at the tumor center. These appeared higher in lower lung densities (lung heterogeneities effect) with the 6X‐FF beams (see Fig. 3).

For the same planning objectives, optimization parameters, and similar target coverage, very small, yet statistically significant differences were observed for all normal lung parameters including V_20Gy_. These values were uniformly lower with clinical 6X‐FFF beams compared to 6X‐FF beams (see *P*‐values in Table 3).

**Table 3:** Normal lung dose statistics between clinical 6X‐FFF and 6X‐FF VMAT plans for all 13 lung SBRT patients. Mean ± standard deviation (range) and *P*‐values were presented.

OAR	Parameters	6X‐FF	6X‐FFF	*P*‐value
Lungs minus ITV	V_20Gy_ (%)	0.6 ± 0.4 (0.1–1.6)	0.6 ± 0.4 (0.1–1.5)	***P = 0.004***
	V_10Gy_ (%)	2.7 ± 1.8 (0.6–6.3)	2.6 ± 1.7 (0.6–6.0)	***P = 0.002***
	V_5Gy_ (%)	6.8 ± 3.7 (2.0–15.0)	6.4 ± 3.5(1.7–14.5)	***P = 0.015***
	MLD (Gy)	1.2 ± 0.6 (0.6–2.4)	1.2 ± 0.5 (0.5–2.3)	***P = 0.002***
	Maximal dose to 1000 cc of lung (Gy)	0.9 ± 1.0 (0.1–3.1)	0.8 ± 0.8 (0.1–2.5)	n. s.

ITV, internal target volume; n. s., not significant; OAR, organs‐at‐risk; SBRT, stereotactic body radiation therapy.

The statistical significance at *P *< 0.05 is bold.

A comparison of other OAR dosimetric parameters for 6X‐FFF and 6X‐FF plans for all 13 lung SBRT patients is presented in Table 4. Critical organs such as spinal cord (D_max_, and D_0.35cc_), heart (D_max_ and D_15cc_), esophagus (D_max_ and D_5cc_), ribs (D_max_ and D_1cc_), and skin (D_max_ and D_10cc_) were evaluated per SBRT protocol guidelines. It was observed that the volumetric dose differences to the heart, esophagus, and ribs were statistically significant (see *P*‐values in Table 4) between the two plans. Overall, the doses with 6X‐FF plans were higher by 1–15% for most of the critical organs, suggesting that the average values of absolute dose differences could be higher with 6X‐FF plans (of the order of 1.0 Gy) compared to clinical 6X‐FFF plans. However, we predict the difference will not be clinically significant.

**Table 4:** Average dose statistics (mean ± SD) for 6X‐FFF and 6X‐FF VMAT plans for all 13 lung SBRT patients.

Dose to OAR	Parameters	6X‐FF	6X‐FFF	*P*‐value
Spinal cord (Gy)	D_max_	3.5 ± 2.6 (0.2–8.6)	3.4 ± 2.5 (0.1–7.8)	n. s.
	D_0.35cc_	3.2 ± 2.3 (0.1–7.6)	3.1 ± 2.3 (0.1– 6.9)	n. s.
Heart/pericardium (Gy)	D_max_	7.9 ± 4.8 (0.2–18.0)	7.7 ± 4.6 (0.1–17.6)	n. s.
	D_15cc_	3.8 ± 2.4 (0.1–9.3)	3.7 ± 2.3 (0.1–9.0)	***P = 0.02***
Esophagus (Gy)	D_max_	4.0 ± 2.0 (0.2–7.0)	3.7 ± 1.9 (0.2–6.7)	***P = 0.01***
	D_3cc_	2.3 ± 1.5 (0.1–4.7)	2.1 ± 1.4 (0.1–4.5)	***P = 0.04***
Ribs (Gy)	D_max_	22.2 ± 6.8 (12.6–31.7)	21.6 ± 6.8 (11.4–31.5)	***P = 0.001***
	D_1cc_	16.9 ± 4.4 (10.0–24.3)	16.5 ± 4.3 (9.4–24.0)	***P = 0.01***
Skin (Gy)	D_max_	8.9 ± 3.0 (4.5–15.2)	9.0 ± 2.6 (5.5–14.5)	n. s.
	D_10cc_	4.7 ± 1.7 (2.8–7.2)	4.7 ± 1.6 (2.9–7.9)	n. s.

n. s., not significant; SBRT, stereotactic body radiation therapy.

The statistical significance at *P* < 0.05 is bold.

### 3.B Treatment delivery parameters

For the given lung SBRT plan, the total number of MU did not change significantly while using 6X‐FF vs 6X‐FFF beams for plan optimization. This suggests that both plans had similar plan complexity providing similar MF.

**Figure 4:**
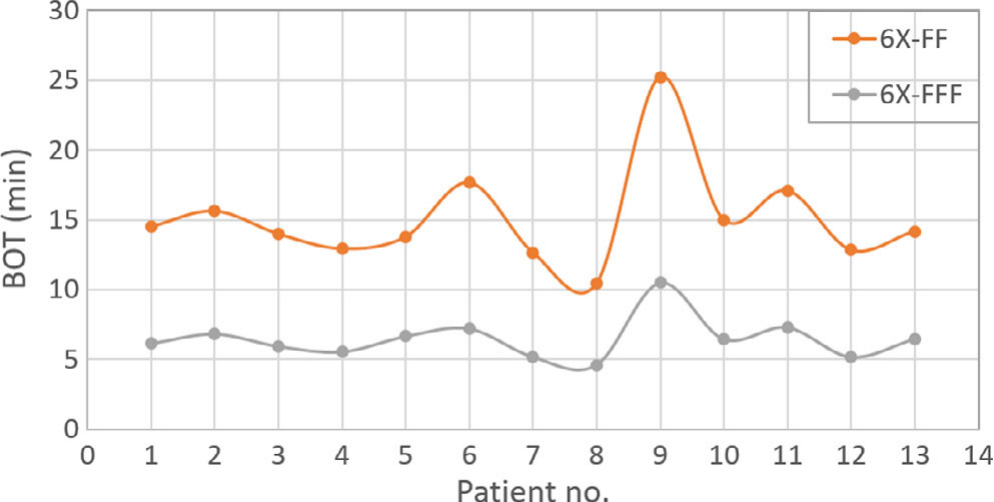
Total BOT on a per‐patient basis, for all 13‐lung SBRT patients treated with a single dose of 30 Gy. The mean value of total BOT was 6.5 ± 1.5 min (with 6X‐FFF) compared to 15.1 ± 3.6 min (with 6X‐FF) showing an on average improvement by a factor of approximately 2.5. BOT, beam‐on time; SBRT, stereotactic body radiation therapy.

However, the average BOT was 6.5 ± 1.5 min (range, 4.6–10.5 min) for 6X‐FFF plans and 15.1 ± 3.6 min (range, 10.41–25.22 min) for 6X‐FF plans, showing a significant difference in treatment time (see p‐value in Table 5). The BOT for 6X‐FF vs 6X‐FFF plans on a per‐patient basis is also shown in Fig. 4.

**Table 5:** Comparison of average values of treatment delivery parameters (and range) between clinical 6X‐FFF and re‐optimized 6X‐FF plans for all 13 lung SBRT patients.

Parameters	6X‐FF	6X‐FFF	*P*‐value
Total monitor units (MU)	9034 ± 2159 (6245–15131)	9040 ± 2045 (6435–14684)	*n. s.*
Modulation factor (MF)	3.0 ± 0.72 (2.1–5.04)	3.0 ± 0.68 (2.15–4.89)	*n. s.*
Beam‐on time, BOT (min)	15.1 ± 3.6 (10.41–25.22)	6.5 ± 1.5 (4.6–10.5)	***P < 0.001***

n. s., not significant; SBRT, stereotactic body radiation therapy.

## 4 DISCUSSION

**Figure 5:**
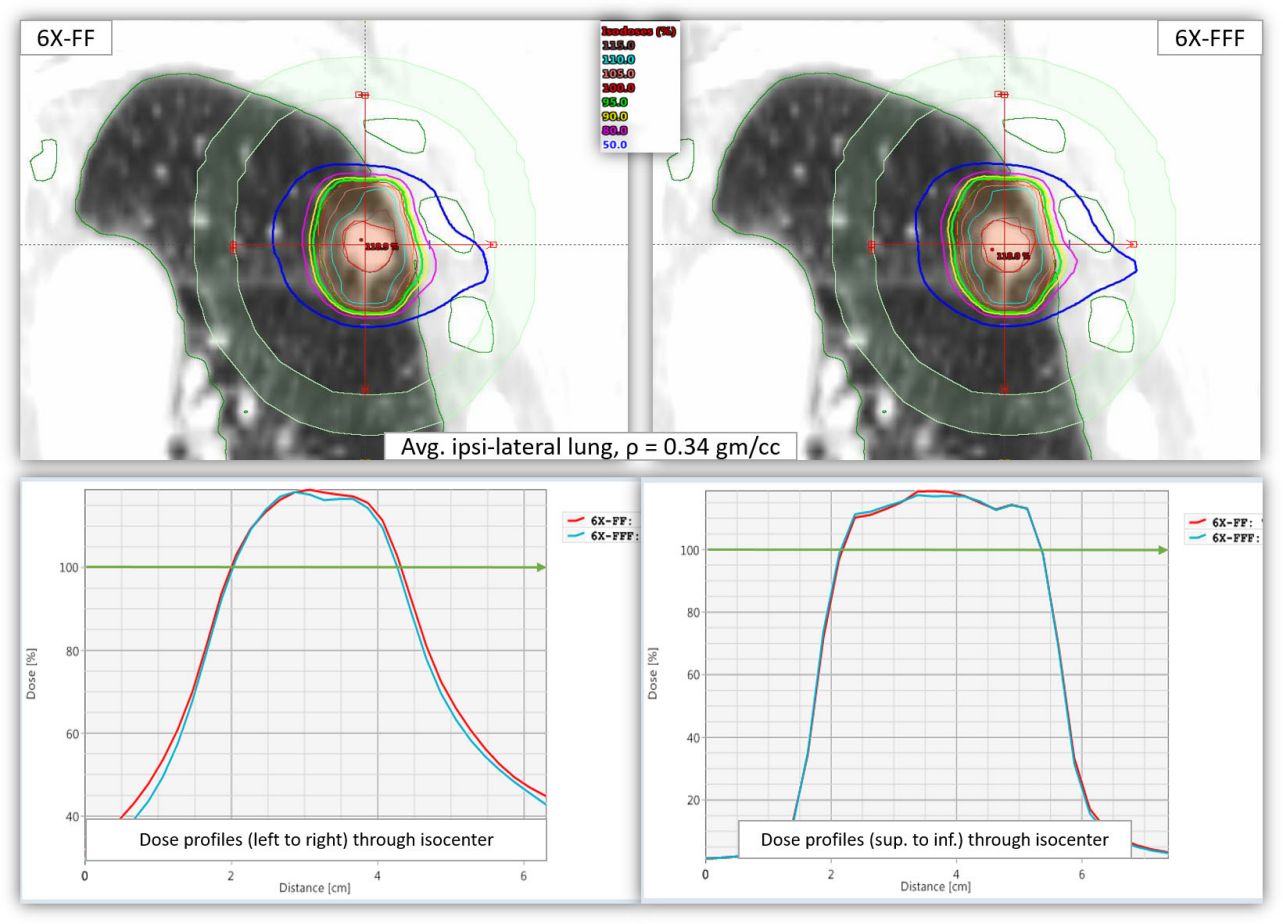
Upper panel: Dose distributions from 6X‐FF (left) and 6X‐FFF (right) in coronal views for another example patient (patient #10) who had the highest average ipsilateral lung density of 0.34 gm/cc. The PTV measured 11.0 cc and was located in the left mid‐lung adjacent to the chest wall. Ribs, normal lung contour, and D2cm ring are shown. Lower panel: Corresponding radial dose profiles drawn through the isocenter location. Bottom left shows the dose profiles from left to right and the bottom right shows the dose profiles from superior to inferior directions (within D_2cm_). The green lines on each graph show that both plans were normalized such that 95% of target coverage received 100% of the prescription dose. For similar target coverage the ITV and out‐of‐fields dose (intermediate dose‐spillage and dose to the OAR) were comparable with both plans. ITV, Internal target volume; OAR, organs‐at‐risk; PTV, planning target volume.

We have presented clinical case studies of radiosurgical dose distributions near the interface between the tumor and lung parenchyma using actual clinical 6X‐FFF plans and traditional 6X‐FF plans for a single‐dose SBRT lung treatment of 30 Gy. In general, 6X‐FFF beams provided dosimetrically superior treatment plans with tighter intermediate dose‐spillage, lower dose to OAR, and much faster treatment delivery (see Tables 2, 3, 4, 5). The tighter dose distributions with 6X‐FFF beams was due to its unique beam profile, softer energy spectrum, and smaller out‐of‐field scatter and leakage characteristics compared to traditional 6X‐FF beams. This effect was much more prominent for the island tumors and increased as a function of lower ipsilateral lung density as shown in Fig. 2. However, insignificant differences between 6X‐FFF and 6X‐FF dose distributions (see Fig. 5) were observed for patient #10. This patient showed the highest average ipsilateral lung density (0.34 gm/cc) and a tumor located adjacent to the chest wall, which suggested that traditional 6X‐FF beams provided a similar dosimetric outcome.

Potential concerns for lung SBRT treatments are low dose‐spillage in the chest wall and ribs, 29, 30, 31 normal lung dose (V_20Gy_, V_10Gy_ and V_5Gy_)^32^, ^33^, ^34^, and higher dose to skin.^35^ For instance, Pettersson and his colleagues 29reviewed 68 NSCLC patients treated via SBRT with 45 Gy in three fractions. Thirty‐three patients had complete clinical follow‐up and radiographic follow‐up exceeding 15 months. Among these, 13 ribs fractures were found in seven patients. In their study, the logistic dose–response curve showed that the risk of radiation‐induced rib fractures following lung SBRT treatments were related to the dose to 2 cc of the rib. For the median follow‐up of 29 months, they showed that the 2 cc of rib receiving (3 × 9.1 Gy) and (3 × 16.6 Gy) had 5% and 50% chances of rib fracture, respectively. In the current study, utilizing 6X‐FFF beams for VMAT planning, all OAR metrics including rib, lung, and skin were lower compared to traditional 6X‐FF beams. Furthermore, all OAR dose metrics were well below the RTOG requirement, suggesting a low probability of acute or late toxicities.

Dose discrepancies due to interplay effects can be concerning for single‐dose SBRT lung treatments with FFF‐VMAT plans. Changes in breathing patterns along with the MLC modulation, gantry rotation, and dose‐rate changes are less likely to average out with a relatively shorter beam‐on time. However, it has been shown in the previous study (with 2400 MU/min) that the interplay effect causes clinically insignificant dose blurring (<3.0%) when using two or more arcs.^36^Our noncoplanar VMAT lung SBRT plans (with 1400 MU/min) uses 3–6 partial arcs, therefore we do not expect clinically significant dose blurring due to MLC interplay. The change in respiratory patterns between the CT simulation and the time of treatment has also been previously studied. 37, 38, 39Although, it has been reported that there were only small changes (within ± 3 mm) due to intrafractional and interfractional motion in lung SBRT treatments, the mean patient setup time from tumor localization to the end of treatment CBCT scan was about 40 min.^39^ It was suggested that an isotropic 5‐mm PTV margin around the ITV (similar to our PTV margin) was sufficient to address the potential motion errors. In this study, our average beam on time was 6.5 min for a single dose of lung SBRT treatment. This potentially decreased the variation of intrafraction motion error due to coughing or pain making a geographic miss less likely and improving the patient stability as well as the clinic workflow.

In summary, each 6X‐FFF and 6X‐FF plan was rigorously evaluated using the dosimetric parameters listed in the Tables 2, 3, 4, 5. All parameters were deemed acceptable per SBRT protocol suggesting that 6X‐FFF plans are dosimetrically superior to 6X‐FF plans. Furthermore, 6X‐FFF plans would also deliver much faster lung SBRT treatments which would potentially improve patient compliance and clinic efficiency. While evaluating the target coverage and OAR doses as a function of average ipsilateral lung density, it was observed that the island tumors with surrounding ipsilateral low lung density showed much higher variation between the modalities (6X‐FFF vs 6X‐FF plans) compared to lesions located near the chest wall with a higher ipsilateral lung density; suggesting that FFF‐beam improves dose coverage at tumor–lung interface. Dose‐limiting toxicity after hypofractionated dose‐escalated radiotherapy in NSCLC patients is still an issue in lung SBRT treatment.^40^ Utilizing 6X‐FFF beams for VMAT SBRT lung planning may potentially reduce dose to OAR, help enhance dose to tumor peripheries, and deliver much faster treatments. However, while optimizing VMAT SBRT lung plans, planners are advised to pay special attention to the ipsilateral lung density and the tumor location on a per‐patient basis as a function of beam modality.

## 5 CONCLUSION

For our 30 Gy single‐dose lung SBRT treatments, 6X‐FFF plans showed dosimetrically superior isodose distributions, lower OAR doses, and much faster treatment deliveries compared to traditional 6X‐FF plans. Additionally, the isodose distributions were significantly affected by the ipsilateral lung density and tumor location as a function of beam modality. The dose enhancement at the tumor periphery was achieved by prescribing dose at the tumor margin (rather than prescribing dose at the tumor center) in addition to using 6X‐FFF beams. Given that FFF‐beam are already widely available in clinics, we hope this study will help in implementation of 6X‐FFF beams for fast, effective, and safe treatment of NSCLC patients treated with SBRT. Clinical follow‐up results of these single‐dose SBRT lung patients are underway.

## CONFLICT OF INTEREST

None.
